# Hierarchical ZIF-8 Materials via Acid Gas-Induced Defect Sites: Synthesis, Characterization, and Functional Properties

**DOI:** 10.1021/acsami.3c08344

**Published:** 2023-08-18

**Authors:** Arvind Ganesan, Johannes Leisen, Raghuram Thyagarajan, David S. Sholl, Sankar Nair

**Affiliations:** † School of Chemical & Biomolecular Engineering, Georgia Institute of Technology, Atlanta, Georgia 30332, United States; ‡ School of Chemistry & Biochemistry, Georgia Institute of Technology, Atlanta, Georgia 30332, United States; § Oak Ridge National Laboratory, Oak Ridge, Tennessee 37830, United States

**Keywords:** MOFs, acid gas, defects, hierarchical, Knoevenagel

## Abstract

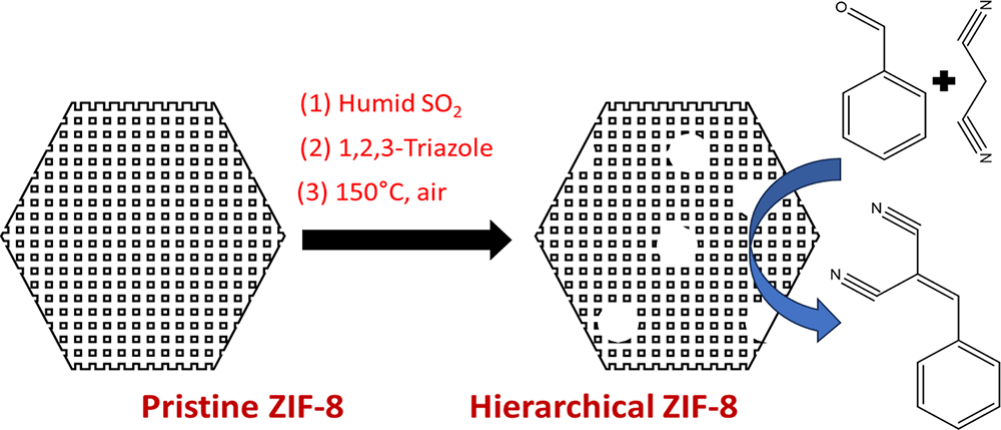

Microporous metal-organic frameworks (MOFs) have been widely studied for molecular separation and catalysis. The uniform micropores of MOFs (<2 nm) can introduce diffusion limitations and render the interiors of the crystal inaccessible to target molecules. The introduction of hierarchical porosity (interconnected micro and mesopores) can enhance intra-crystalline diffusion while maintaining the separation/catalytic selectivity. Conventional hierarchical MOF synthesis involves complex strategies such as elongated linkers, soft templating, and sacrificial templates. Here, we demonstrate a more general approach using our controlled acid gas-enabled degradation and reconstruction (Solvent-Assisted Crystal Redemption) strategy. Selective linker labilization of ZIF-8 is shown to generate a hierarchical pore structure with mesoporous cages (∼50 nm) while maintaining microporosity. Detailed structural and spectroscopic characterization of the controlled degradation, linker insertion, and subsequent linker thermolysis is presented to show the clustering of acid gas-induced defects and the generation of mesopores. These findings indicate the generality of controlled degradation and reconstruction as a means for linker insertion in a wider variety of MOFs and creating hierarchical porosity. Enhanced molecular diffusion and catalytic activity in the hierarchical ZIF-8 are demonstrated by the adsorption kinetics of 1-butanol and a Knoevenagel condensation reaction.

## Introduction

Metal-organic frameworks (MOFs) provide excellent opportunities for size and shape selective separation.^1−6^ The microporous structure of MOFs can be used in molecular recognition and separation.^7^ However, the uniformly small pores (<2 nm) lead to slow diffusion rates, and reduced access into MOF crystals.^1,8^ Hierarchical pores with interconnected mesopores (2–50 nm) and nanopores (<2 nm) can circumvent these limitations,^9^ potentially extending the application of MOFs in catalysis, adsorption, and chemical sensing. Several efforts have focused on the synthesis of hierarchical zeolites to substantially resolve the low masstransfer, deactivation, and reduced reactivity of bulky substrates.^10−12^ Similarly, the potential applications of hierarchical MOFs such as separation,^13−15^ catalysis,^16−18^ adsorption,^19,20^ gas storage,^21^ and biomolecular carriers^22−28^ would benefit from the development of strategies to synthesize hierarchical MOFs.

Several efforts have targeted creating mesopores or hierarchical pores in MOFs. Reticular synthesis^29^ with longer linkers^30,31^ is one strategy, but increased flexibility from longer organic linkers can result in interpenetrated structures, reducing the effective pore size.^32^ Another interesting method is templated synthesis using surfactants and micellar structure directing agents (SDAs).^8,14,33^ Unlike the use of SDAs in zeolites (wherein the SDA can be removed by calcination), MOFs require the use of soft templating^15−17^ and careful solvent exchange to maintain porosity.^16,17,34,35^ Alternatively, mild acids,^34^ mineral acids,^35^ or water have been used as post-synthetic etchants to generate mesopores. This liquid-assisted etching is diffusion-dominated, potentially leading to non-uniform etching with core-shell morphologies.^34,36^ Other strategies include the use of expensive switchable solvent^37^ and supercritical CO_2_.^21^ These strategies are complex to operate and difficult to extend to other MOFs.^38^ Modulating ligands,^39^ mono-dentate linkers,^40^ and defect engineering^41^ are also being studied to induce mesopores in MOFs.

Mixed-linker materials offer another approach to creating mesopores in MOFs by incorporating ‘pro-labile’ linkers that are selectively susceptible to the subsequent generation of hierarchical porosity.^38,42,43^ However, almost all the early work focuses on the denovo synthesis of mixed-linker MOFs^38,42,43^ only enabling gradual increments in pore size due to the merging of cages formed by the removal of linkers. Typical in-situ mixed-linker syntheses are governed by thermodynamic factors that make it difficult to create mesopores in a controlled fashion.^44^ Post-synthetic linker insertion in ZIFs is diffusion limited and leads to core-shell distributions.^45^ An alternative is the so-called solvent-assisted crystal redemption (SACRed)^46^ method, which can be used to selectively incorporate non-native linkers.^47^ SACRed exploits the inherent instability of many MOFs in acid gases^48−50^ and forms interesting hybrids from defective or degraded MOFs.^47^ By decoupling crystal formation from linker insertion, SACRed can circumvent many of the thermodynamic and kinetic barriers experienced by the other synthesis strategies.

Our recent work has shown the generalizability of the SACRed method to multiple acid gases (humid/dry SO_2_ and NO_2_) and a range of MOFs (ZIF-8, ZIF-90, ZIF-71, UiO-66, and ZIF-67).^51^ Furthermore, in this work, SACRed has been demonstrated on ZIFs with particle sizes ranging from ∼350 nm (ZIF-71) to 150 μm (ZIF-90) with significant recovery^51^ of crystallinity and porosity (Figure S1a,b, Supporting Information). Combining this with selective linker thermolysis^42,43^ would provide a more general strategy for the synthesis of hierarchical MOFs. The effective use of defects to design and synthesize hybrid materials requires an understanding of the type, distribution, and correlation of such defects. In addition to our recent computational^52^ and modeling of high-resolution x-ray diffraction data^53^ showing the clustering of acid gas-induced defects, experimental validation is essential. Although characterization techniques such as PXRD, HRTEM, FTIR, and EXAFS have been useful in observing the structural effects and composition of defects,^54^ the distribution and interactions of intracrystalline defects are less explored.

**Figure 1:**
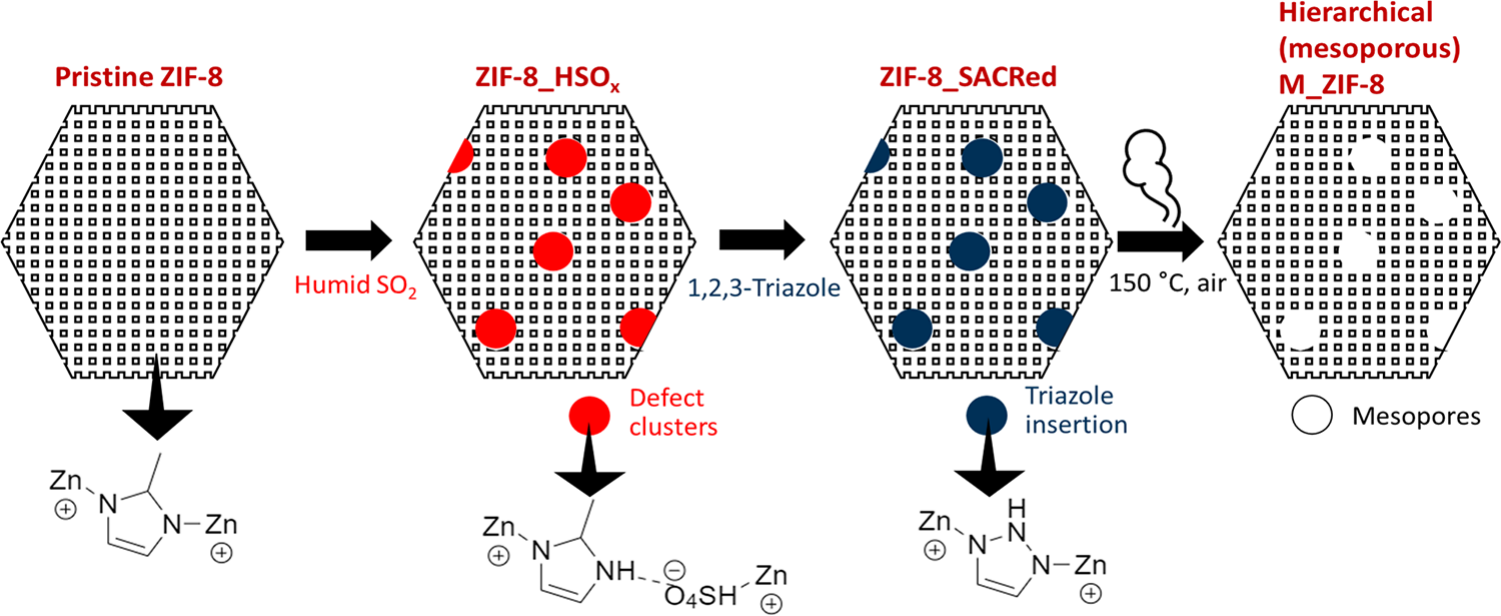
Schematic showing the synthesis of hierarchical (mesoporous) ZIF-8 from pristine (microporous) ZIF-8 by introduction of acid gas induced defects, non-native linker insertion, and non-native linker thermolysis.

In this paper, we report a significant extension of the SACRed method to create hierarchal porosity in MOFs. Figure 1 shows a schematic of the method. Specifically, we hypothesized that SACRed provides a route to incorporate clusters of pro-labile linkers in template MOFs through controlled exposure to acid gases and subsequent insertion of a pro-labile linker. The selective removal of these clusters of pro-labile linkers can then enable the controlled formation of larger (meso- or macro-) pores in microporous MOFs. We explore this strategy by synthesizing and characterizing a hierarchical ZIF material starting from ZIF-8. We focus on probing the clustering of defects due to acid gas exposure with double-quantum spin-exchange nuclear magnetic resonance (DQ-NMR) measurements and the selective replacement of these defects with a pro-labile triazole linker. We show the successful formation of mesopores by linker thermolysis while retaining the template ZIF-8 structure and morphology with a suite of qualitative and quantitative characterization techniques. We also demonstrate enhancement of the catalytic reaction kinetics of a Knoevenagel condensation, due to improved mass transfer in the hierarchical ZIF-8 material relative to a purely microporous ZIF-8 material.

## Experimental Methods

### Materials

Zinc nitrate hexahydrate (Sigma Aldrich), 1,2,3-triazole (Triazole) (Sigma Aldrich), 2-methylimidazole (2-MeIm) (Sigma Aldrich), sodium formate (Alfa Aesar), methanol (VWR), malononitrile (Sigma Aldrich), benzaldehyde (Sigma Aldrich), tetrahydrofuran (THF) (VWR), deuterated 2-methylimidazole (CDN Isotopes), benzylidenemalononitrile (MilliporeSigma), and ethylacetate (VWR) were used as received. Deionized water from an EMD Millipore water purification system, 1000 ppm SO_2_ with balance N_2_, ultra-high purity hydrogen, helium, and air (76.5–80.5% N_2_, 19.5–23.5% O_2_) from Airgas were used.

### ZIF Synthesis

ZIF-8 was synthesized with a modified procedure from Zhang et al.^55^ Typically, 0.297 g of Zn(NO_3_).6H_2_O was dissolved in 20 mL of methanol. 0.164 g of 2-MeIm was dissolved in 20 mL of methanol with 0.269 g of HCOONa. The metal solution was added to the linker solution and transferred to a Teflon liner. The liner was then sealed inside a Parr Instruments stainless steel autoclave and incubated at 363 K for 48 h. The crystals were centrifuged at 8500 rpm for 5 mins and washed thrice with fresh methanol. The crystals were air dried at 333 K. Dried crystals were then degassed at 453 K in a vacuum overnight. Deuterated ZIF-8 (ZIF-8-d) was synthesized as mentioned above by replacing the protonated linker with the deuterated linker.

### Characterization

Degassed ZIF-8 samples (pristine, acid gas exposed, and hierarchical) were characterized to determine their physical and chemical properties. An PANalytical X’Pert Pro diffractometer (CuKα source, λ = 0.1541 nm) was operated at 45 kV and 40 mA for collecting powder X-ray diffraction (PXRD) patterns with a scan time of 12 step/sec and a step size of 0.0167° 2θ over the range of 5–50° 2θ. The Brunauer-Emmett-Teller (BET) surface area and t-plot pore volume were evaluated from N_2_ adsorption isotherms at 77 K obtained with Micromeritics Tristar II 3020 and MicroActive V3 analyzers. The BET parameters were optimized for each N_2_ isotherm using the Rouquerol criteria.^56^ SEM measurements were carried out with a Hitachi SU 8010 scanning electron microscope. Water vapor and 1-butanol adsorption experiments were performed using a dynamic vapor sorption device, DVS-Advantage (Surface Measurement Systems). All samples were pretreated at 100 °C for 6 h under nitrogen flow and cooled to 30 °C. Then the samples were exposed to water (or 1-butanol) vapor at various partial pressures between 0 and 95% relative humidity. Solution NMR measurements were performed with an Avance III 400 MHz and CD_3_COOD as the solvent. Solid-state NMR measurements were performed with a Bruker Avance III HD NMR spectrometer operating at a ^1^H frequency of 500 MHz. The Magic Angle Spinning (MAS) experiments were conducted on 3.2 mm o.d. MAS rotors using a spinning speed of 23 kHz. 1D spectra were recorded using a simple, single-pulse excitation with π/2 pulse length of 2.5 μs and a repetition delay of 1 s. A good signal-to-noise ratio could be achieved with 128 scans. Single-quantum-double-quantum correlation experiments were conducted using the BABA-2 sequence^57^ under the conditions of MAS at 23 kHz. The sequence is based on the recoupling of dipolar interactions during 2 rotor cycles. For all samples investigated in this study, the maximum signal intensity of the measured double-quantum signals was achieved during this minimum recoupling cycle. The combustion thermogravimetric analysis (TGA) was conducted with a TA Instruments TGA550. The furnace temperature was gradually increased from room temperature to 125 °C under 100 mL/min of nitrogen (N_2_) and was then further raised to 700 °C under 100 mL/min of ultra-zero grade air. The temperature ramp rate for both steps was set at 10 °C/min.

### Controlled Acid Gas Exposure

Activated ZIF-8 samples were exposed in a custom-built system for the acid gas exposure of MOFs. Acid gas was sourced from lecture bottles with an acid gas concentration of 1000 ppm SO_2_ in N_2_. The custom-built system is hosted inside a fume hood with acid gas lecture bottles to prevent any leaks. 2 Dräger PAC 7000 SO_2_ personal monitors (inside and outside the fume hood) to ensure a safe working environment. Humidity is generated with ultra-high purity air saturated in a humidity bottle from Fuel Cell Technologies Inc. A mixture of these streams is fed into an exposure unit. The samples were exposed to a total dose of 75 ppm-days of SO_2_ (concentration of SO_2_ × time) at 85% Rh and room temperature.

### Solvent-Assisted Crystal Redemption (SACRed)

Around 150mg of degraded MOF samples were treated with 0.25 M solutions containing the fresh 1,2,3-triazole (triazole) linker dissolved in methanol. The degraded ZIF-8 samples were dispersed in 40 mL of fresh triazole solution inside Teflon-lined Parr autoclaves and incubated in an oven without rotation for 6 h at 363 K. The recovered crystals were separated from the mother liquor and washed thrice with fresh methanol. Overnight linker thermolysis was performed at 423 K in air. Dried samples were vacuum activated at 453 K overnight.

### Diffusivity Calculations

The Fickian transport diffusion coefficient *D* of 1-butanol in the ZIF-8 crystals is determined by fitting the fractional mass uptake from kinetic measurements to^58^
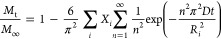
1where *X_i_* is the volume fraction of the crystal with particle radius *R_i_*. Using the adsorption isotherm data, the loading dependence of the estimated transport diffusivity can be decoupled by estimating the corrected diffusivity *D*_0_ using:^59^
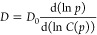
2where *p* is the pressure and *C*(*p*) is the adsorption loading. We fit the adsorption isotherm to the Langmuir model:
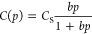
3where *C*_S_ is the saturation loading and *b* is the Henry’s constant. Combining eqs 2 and 3:
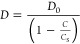
4

### Catalytic Activity Measurements

The catalytic activity of the degassed MOF samples was tested with the Knoevenagel condensation of benzaldehyde and malononitrile described by Shen et al.^17^ Typically, 6.6mg of MOF sample was mixed in 5 mL of THF with 0.201 g of malononitrile and 0.251 g of benzaldehyde. The reaction mixture was stirred at room temperature for a preset amount of time, and ethyl acetate was used to quench the reaction. The reaction mixture was filtered with a 0.2 μm PTFE filter and analyzed with a Shimadzu GC 2010 Plus.

### Batch Adsorption Measurements

Typically, 6.6 mg of MOF sample was mixed in 5 mL of THF. 30 mg of benzaldehyde or benzylidenemalononitrile was then added to the dispersed MOF solution. After stirring the mixture at room temperature for a preset amount of time, the supernatant was filtered with a 0.2 μm PTFE filter and analyzed with a Shimadzu GC 2010 Plus. The Fickian transport diffusion coefficient *D* of benzaldehyde and benzylidenemalononitrile in the ZIF-8 crystals is determined by fitting eq 4 to the fractional mass uptake data from the adsorption measurements.

## Results and Discussion

**Figure 2:**
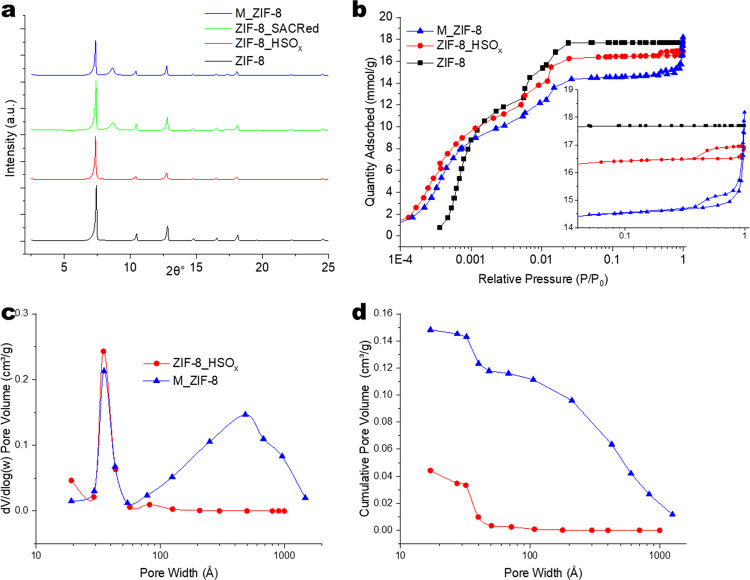
(a) PXRD patterns of ZIF-8, ZIF-8_HSO_*x*_), ZIF-8_SACRed, and Mesoporous ZIF-8 (M_ZIF-8), stacked with a vertical offset; (b) N_2_ adsorption isotherms of ZIF-8, ZIF-8_HSO_*x*_, and M_ZIF-8, (b inset) *P*/*P*_0_ range of 0.04–1 showing hysteresis loops; (c) mesopore size distributions and (d) cumulative mesopore volumes of ZIF-8_HSO_*x*_ and M_ZIF-8.

The crystallinity of pristine ZIF-8, acid gas exposed ZIF-8_HSO_*x*_, triazole-inserted ZIF-8_SACRed, and mesoporous M_ZIF-8 was characterized by PXRD (Figure 2a). The crystallinity and SOD topology of ZIF-8 are maintained throughout the processes of acid gas exposure, selective insertion, and thermolytic removal of triazole. A new peak at 2θ° ∼ 8.6° was observed in ZIF-8_SACRed and M_ZIF-8 materials, similar to our earlier work with ZIF-8-7 mixed-linker hybrid materials prepared via SACRed.^47^ This peak arises due to minor changes in crystal symmetry during the process of selective linker replacement. These effects are absent in ZIF-8_HSO_*x*_ due to the formation of amorphous domains with diffuse scattering.^51,53^ The small peaks ∼8.6° (ZIF-8_SACRed and M_ZIF-8) are introduced with the incorporation of triazole linkers and the associated regeneration of crystal order. On the other hand, significant peak broadening and a change in crystal topology have been observed after the post-synthetic insertion of triazole in ZIF-8.^60^

Figure 2b shows the N_2_ adsorption isotherms of ZIF-8, ZIF-8_HSO_*x*_, and M_ZIF-8 measured at 77 K. A typical Type I microporous isotherm is obtained for ZIF-8, confirming the presence of a highly crystalline microporous structure. On the other hand, a Type IV isotherm is observed for both ZIF-8_HSO_*x*_ and M_ZIF-8, indicating the formation of mesopores with acid gas exposure and the pro-labile linker thermolysis. The Type IV (hysteresis H1) isotherm seen in M_ZIF_8 (Figure 2b inset) typically represents cage-like porosity.^61^ Both ZIF-8_HSO_*x*_ and M_ZIF-8 showed N_2_ adsorption at lower relative pressures than pristine ZIF-8, due to the introduction of mesopores that increase access to the micropore structure (Figure 2b). The pro-labile triazole linker in ZIF-8_SACRed is prone to partial thermolysis during the degassing step under vacuum. This prevents us from reliably using N_2_ adsorption to unambiguously assess the porosity of this material. As shown in our previous work, the introduction of defects (dangling acid gas-linker complexes) in ZIF-8_HSO_*x*_ after acid gas exposure causes structural distortions at the unit cell level and the presence of flexible hydrogen bonds.^47^ The M_ZIF-8 shows significant mesoporosity with the presence of a hysteresis loop in the N_2_ adsorption isotherms for *P*/*P*_0_ ∼ 0.04–1 (Figure 2b inset).

The mesopore size distributions (Figure 2c), as well as the hysteresis behavior (Figure 2b inset), suggest the formation of small mesopore regions (∼3.5 nm) that span 2–3 unit cells (a single ZIF-8-unit cell cage has a size of 1.4 nm). These regions are present in the acid gas-exposed (ZIF-8_HSO_*x*_) and are retained in the hierarchical M_ZIF-8 after burnout of 1,2,3-triazole. The M_ZIF-8 material also shows a second, broad mesopore distribution with an average pore size of 50 nm (Figure 2c), which is created by the burn-out of 1,2,3-triazole. Figure 2d shows the cumulative mesopore volume distribution with BJH desorption analysis for both these materials, with the final M_ZIF-8 material having much higher mesoporosity than the intermediate ZIF-8_HSO_*x*_ material.

Table 1 summarizes the textural characteristics obtained from the N_2_ physisorption isotherms. The pore volume (0.61 cm^3^/g), BET surface area (1884 m^2^/g), and negligible mesoporosity of the starting template material (ZIF-8) are in accordance with the literature.^62,63^ The final hierarchical material M_ZIF-8 shows a large mesopore volume (0.15 cm^3^/g), compared to 0.045 cm^3^/g of mesopore volume formed during the acid gas exposure. Thus, the overall mesoporosity of the M_ZIF-8 material arises from both the acid gas exposure (which creates small 3.5 nm mesopore regions) and the subsequent burn-out of 1,2,3-triazole (which creates larger mesopores). M_ZIF-8 also retains a significant microporous BET surface area and pore volume, implying that the ZIF-8 template retains its overall microporous crystal structure. Furthermore, no large changes were observed (by SEM) in overall crystal size or morphology due to the generation of hierarchical porosity (Figure S1, Supporting Information), although the surface of the M_ZIF-8 crystals showed some pitting during the process.

**Table 1:** Textural Characteristics of ZIF-8, ZIF-8_HSO_*x*_, and M_ZIF-8 Obtained from N_2_ Adsorption Isotherms

material	BET surface area (m^2^/g)	micropore volume (cm^3^/g)	mesopore volume (cm^3^/g)
ZIF-8	1884	0.61	
ZIF-8_HSO_*x*_	1551	0.55	0.045
M_ZIF-8	1372	0.49	0.15

**Figure 3:**
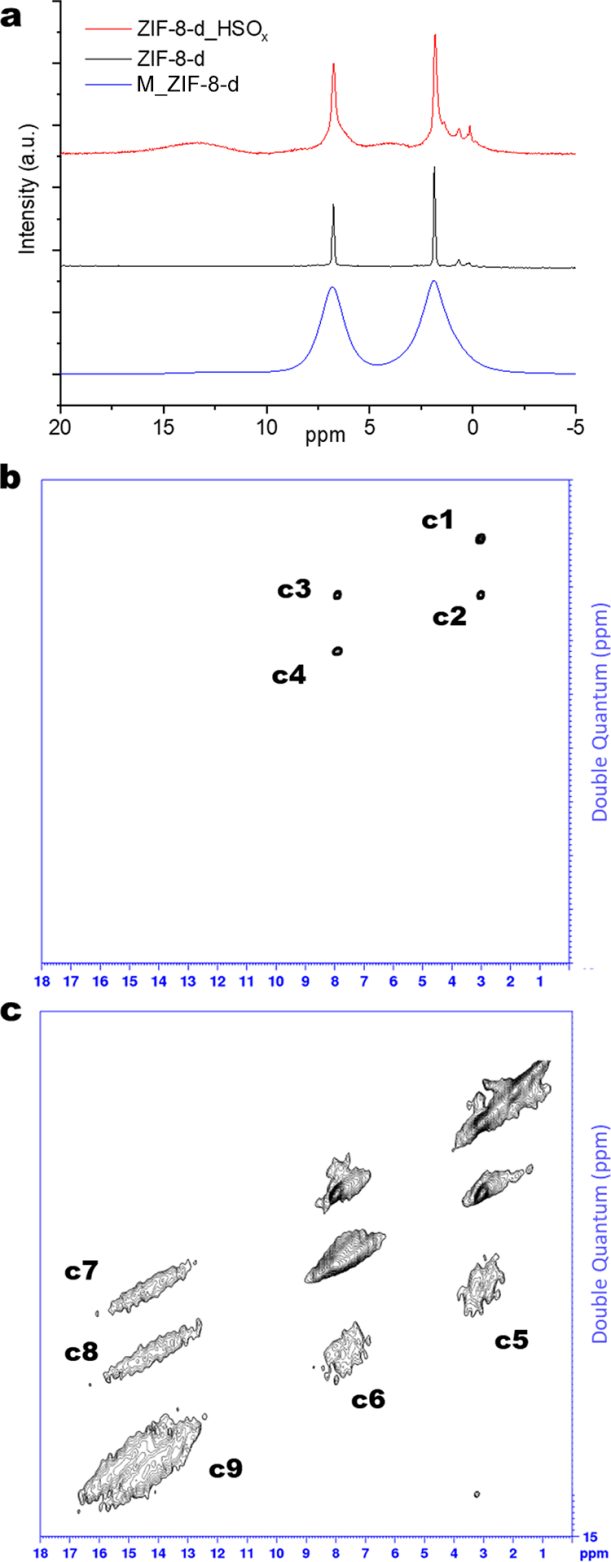
(a) 1-dimensional solid-state ^1^H NMR spectra of ZIF-8, ZIF-8-d, and ZIF-8-d_HSO_*x*_; multi-quantum spin-exchange spectra of (b) ZIF-8-d and (c) ZIF-8-d_HSO_*x*_.

Figure 3 presents the NMR analysis of the above materials. The ^1^H-MAS spectrum of ZIF-8_HSO_*x*_ was found to be nearly identical to those of the pristine ZIF-8, due to the low overall concentration of HSO_*x*_ defects and the broad line shapes. To study the incorporation of HSO_*x*_ defects in ZIF-8, the material was synthesized using highly deuterated (∼97%) 2-methylimidazole linkers. The remaining ∼3% of non-deuterated protons allow readily detectable signals from both the methyl (1.82 ppm) and imidazole (6.85 ppm) protons of the linker in the deuterated MOFs. Figure 3a shows the 1D solid-state ^1^H-NMR spectra of deuterated ZIF-8-d and ZIF-8-d_HSO_*x*_ as well as the non-deuterated ZIF-8_HSO_*x*_. The deuterated materials show significant narrowing of spectral lines in comparison to the fully protonated sample, because the dilution of the ^1^H nuclei leads to the reduction of homonuclear dipolar coupling between the individual sites.^64^ Two new broad peaks are observed in deuterated ZIF-8-d_HSO_*x*_ (Figure 3a), in addition to the methyl and aromatic imidazole proton signals. The broad peak around 4.2 ppm represents atmospheric H_2_O absorbed around the hydrophilic defects in ZIF-8-d_HSO_*x*_. The broad peak around 13.5 ppm corresponds to the HSO_*x*_ proton signal^65^ and shows the ability to observe the proton signals from acid gas-induced defects in MOFs. Based on spectral densities in ZIF-8-d_HSO_*x*,_ the molar ratio of ^1^H originating from HSO_*x*_ relative to ^1^H in the linker is about 1:3. Assuming a 97% degree of linker deuteration (specified in the as-received deuterated 2-methylimidazole), we can quantify the molar ratio of HSO_*x*_ defects to linker molecules to be about 1:20. The comparison of spectra in Figure 3a also shows that the introduction of the acid gas leads to a significant broadening of the linker signals in ZIF-8-d_HSO_*x*_ relative to the narrow peaks seen in pristine ZIF-8-d. This is clear evidence that the previously isolated ^1^H linker sites in the pristine material now have increased dipolar coupling with other ^1^H sites (which must originate from the HSO_*x*_ defects). Based on the estimated HSO_*x*_:linker ratio of 1:20 (i.e., about 5% defects), the broad line shapes provide strong evidence that a significant amount of acid gas-induced defects are distributed spatially in a relatively homogeneous manner within the ZIF-8 crystal, facilitating ^1^H dipolar couplings between the linker protons (∼3% in deuterated linkers) and the acid gas defect protons (∼5%). The defect concentrations obtained from ^1^H NMR are also broadly consistent with those obtained by the elemental analysis of sulfur (S) and nitrogen (N) in our previous work.^66^

Our ability to clearly observe the defects in ^1^H NMR provides an opportunity to quantify their distribution/clustering via the dipolar coupling of protons using two-dimensional single-quantum-multiple-quantum (SQ-MQ) correlation spectroscopy. The advantage of this technique is the ability to detect proximities not only between sites with different chemical shifts/environments but also between sites with identical chemical shifts.^57^Figure 3b shows the ^1^H SQ-MQ-NMR correlation spectrum of deuterated ZIF-8-d. Although minimal proton-proton interactions are anticipated in a ∼97% deuterated sample, a few individual linker molecules would still be highly protonated. The presence of 2-methyl and 4,5-imidazole protons on the same linker molecule leads to characteristic peaks mapping the dipolar coupling in the imidazole linker. The contours b1–b4 represent these spin couplings between methyl-methyl, methyl-imidazole, imidazole-methyl, and imidazole-imidazole respectively on the same linker molecule. It should be noted that the equivalent of the solid-state SQ-MQ NMR experiment is the INADEQUATE experiment in solution NMR,^67^ which is known to detect the coupling of ^13^C sites at a low natural ^13^C abundance of ∼2%.^67^Figure 3c shows analogous SQ-MQ data for ZIF-8-d_HSO_*x*_. Additional peaks (c5–c9) corresponding to the coupling of the acidic (HSO_*x*_) defect with the linker (methyl and imidazole) protons are present, clearly identifying the dangling acid gas-linker complexes^51^ formed due to the acid gas exposure. The contour c9 represents the coupling within a defect cluster. Upon closer inspection, this peak appears to be an overlay of two peaks, while only single peaks are found for c5–c8. We speculate that the structure of the c9 contour is due to a distribution in the sizes and locations of HSO_*x*_ clusters. Some clusters may experience interaction with nearby protonated imidazole linkers, while other clusters may only experience internal interactions between the chemically near-identical HSO_*x*_ defect sites. These observations also provide broad corroboration for our recent computational and modeling studies that predicted clustering of HSO_*x*_ defects in ZIF-8.^52,53^

**Figure 4:**
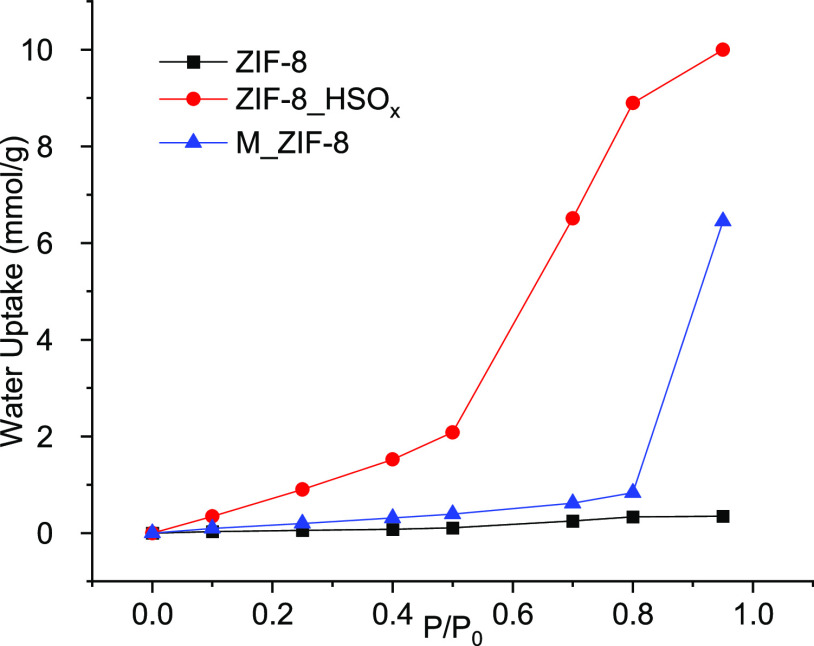
Water adsorption isotherms of ZIF-8, ZIF-8_HSO_*x*_, and M_ZIF-8 at 303 K.

The water adsorption isotherms for ZIF-8, ZIF-8_HSO_*x*,_ and M_ZIF-8 at 303 K are shown in Figure 4. The negligible water adsorption in ZIF-8 is consistent with our earlier observations^68^ and confirms its hydrophobic nature. In contrast, ZIF-8_HSO_*x*_ shows significant water uptake at relative pressures >0.5, consistent with the presence of water signals in the solid-state ^1^H-NMR spectrum (Figure 3a). Protons of water adsorbed directly on the HSO_*x*_ defects are expected to experience rapid exchange with the defect (HSO_*x*_) protons, which would result in a single NMR chemical shift signal for both protons. However, the separate water peak at ∼4.2 ppm observed in Figure 3a indicates the presence of isolated water domains/multi-molecule clusters that are not adsorbed on the HSO_*x*_ defects. This is consistent with our recent computational predictions of water clustering in ZIF-8.^47^ The observed maximum water uptake of ZIF-8_HSO_*x*_ (∼10 mmol/g or ∼ 0.18 cm^3^/g at *P*/*P*_0_ = 0.95) is about one-third of its overall pore volume (Table 1), indicating a partial filling of pores with water. This observation is consistent with the presence of hydrophobic regions and a non-uniform distribution of hydrophilic defects. The final M_ZIF-8 material shows negligible adsorption at *P*/*P*_0_ < 0.80, indicating that the hydrophobicity of ZIF-8 is recovered after selective linker replacement and 1,2,3-triazole thermolysis (burn-out). At the highest relative pressure measured (*P*/*P*_0_ = 0.95), M_ZIF-8 shows significant uptake of water. This is consistent with the condensation of water within the large mesopores of the hierarchical material (Figure 4), which are absent in pristine ZIF-8 and ZIF-8_HSO_*x*_.

**Figure 5:**
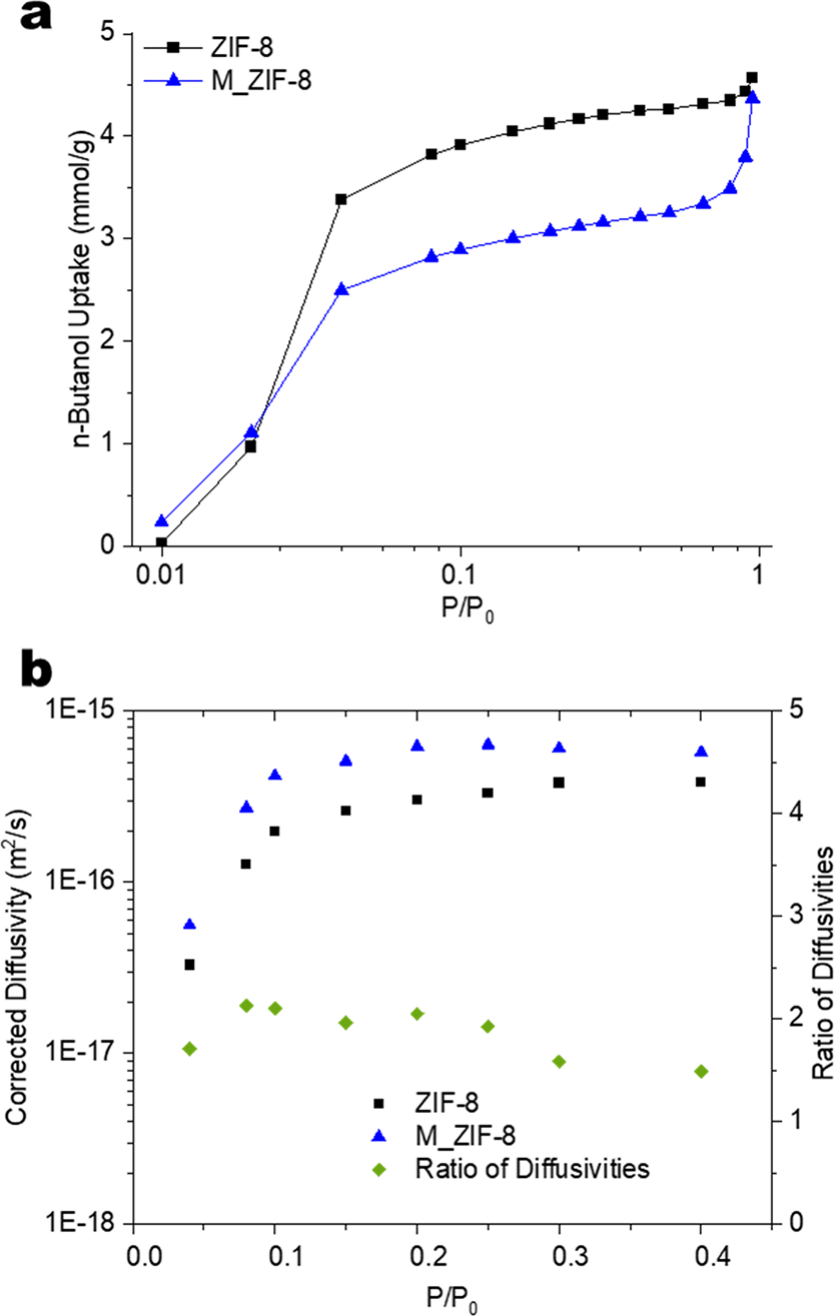
(a) adsorption isotherms of 1-butanol in ZIF-8 and M_ZIF-8 at 303 K, and (b) corrected diffusivities of 1-butanol in ZIF-8 and M_ZIF-8 at 303 K.

Figure 5a shows 1-butanol adsorption isotherms for ZIF-8 and M_ZIF-8. The butanol uptakes of ZIF-8 and M_ZIF-8 at *P*/*P*_0_ < 0.80 show a similar trend, indicating that the overall microporous structure of ZIF-8 is retained in the hierarchical M_ZIF-8. The maximum loading of butanol in M_ZIF-8 at *P*/*P*_0_ = 0.80 is also commensurate with the micropore volume retained in the hierarchical material (Table 1). The significant increase in uptake by M_ZIF-8 starting at *P*/*P*_0_ = 0.80 represents the condensation of butanol in the mesopores. This is not observed in ZIF-8, which only shows a slight increase in uptake near *P*/*P*_0_ ∼ 1 due to capillary condensation in the interstitial spaces between the adsorbent particles. The kinetics of uptake at each pressure (obtained during the isotherm measurements) allow determination of the corrected diffusivity of 1-butanol (see Experimental Methods, eqs 1–4) in the two materials as a function of *P*/*P*_0_. The diffusion characteristics of 1-butanol entering ZIF-8 and M_ZIF-8 are also useful for probing differences between the materials. The corrected diffusivities (Figure 5b) increase with relative pressure for *P*/*P*_0_ < 0.2, given that this region is dominated by strong adsorbate-adsorbent interactions.^69−71^ The diffusivities plateau at higher pressures due to increasing saturation of the pore volume with the 1-butanol adsorbate and the resulting effects of adsorbate-adsorbate interactions. The corrected diffusivities of 1-butanol in M_ZIF-8 are seen to be 50–113% larger than for pristine ZIF-8. The considerably enhanced diffusivity in the hierarchical material can be directly attributed to the presence of mesopores that allow faster access to the microporous regions by reducing the effective diffusion lengths. All the data shown in Figure 5b is in the micropore filling region (*P*/*P*_0_ < 0.80), hence the faster diffusion in M_ZIF-8 is not an effect of condensation in the mesopores. Figure S1 also shows that the crystal sizes of ZIF-8 and M_ZIF-8 are similar, hence crystal size effects do not cause the observed behavior.

**Figure 6:**
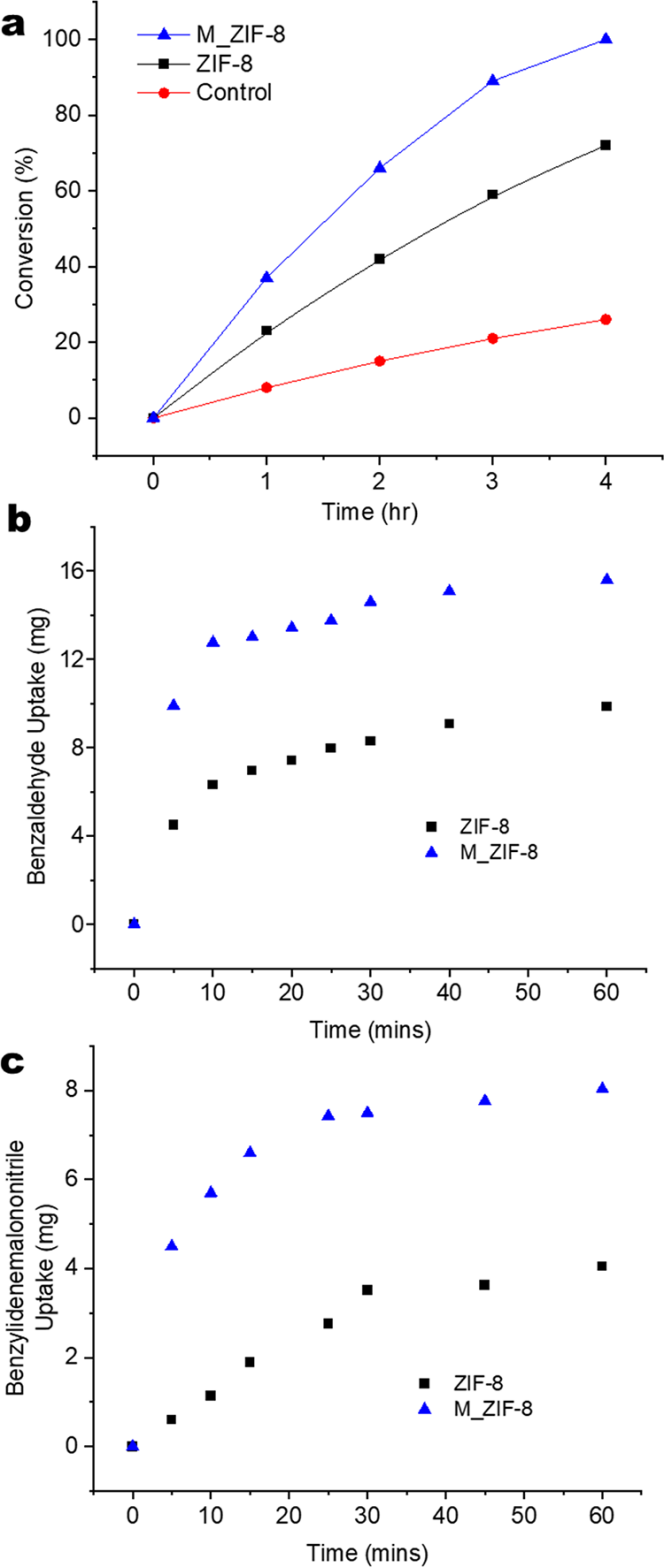
(a) Knoevenagel condensation conversion at 298 K versus time with no catalyst (red), ZIF-8 (black) and M_ZIF-8 (blue), and (b) and (c) batch adsorption of benzaldehyde and benzylidenemalononitrile in ZIF-8 (black) and M_ZIF-8 (blue) respectively.

Figure 6a shows the Knoevenagel condensation kinetics of benzaldehyde and malononitrile into benzylidenemalononitrile (BMN) over ZIF-8 and M_ZIF-8 catalysts in a batch reactor. The mass of both catalysts was identical in the experiments. Similar particle sizes of ZIF-8 and M_ZIF-8 were used in these experiments, so that the reactant/product diffusion length scales are similar in both materials. ZIF-8 shows faster conversion compared to the control (no catalyst), thereby confirming the catalytic activity of the imidazole nitrogen sites, and the reactants and products have access to the micropores of ZIF-8. The M_ZIF-8 catalyst shows considerably faster conversion compared to ZIF-8. Figure S3a shows the TGA curves for ZIF-8 and M_ZIF-8. The overall metal-to-linker ratio remains the same after introduction of mesopores. The solution NMR spectrum of digested M_ZIF-8 (Figure S3b) shows ∼7.5% triazole linkers (mol/mol basis). The TGA and solution NMR data together indicate that there are only ∼3.6% more catalytic N sites in M_ZIF-8 than in ZIF-8. The enhanced conversion in M_ZIF-8 cannot be explained by this minor difference in the overall number of catalytic sites between the two materials. To determine the role of reactant/product diffusion in explaining these results, we measured the adsorption uptake kinetics of BMN (the largest molecule in the reaction) and benzaldehyde (the larger of the two reactants). The single-component uptake experiments were performed in batch mode at 298 K with 30 mg of benzaldehyde, or BMN and 6.6 mg of ZIF-8, or M_ZIF-8. Both materials show significant adsorption of this species (Figure 6b,c) on time scales similar to that of the reaction kinetics, indicating that all the molecular species have access to intracrystalline pores in the two catalysts. M_ZIF-8 has a higher saturation uptake of both molecules than ZIF-8, owing to the additional mesoporosity of the hierarchical material. Figure S2 shows the fits of the fractional mass uptake data to the Fickian diffusion model shown in eq 1. ZIF-8 shows diffusivities of 3.4 × 10^–15^ m^2^/s (benzaldehyde) and 1.7 × 10^–15^ m^2^/s (BMN). M_ZIF-8 clearly shows enhanced diffusivity of benzaldehyde (6.7 × 10^–15^ m^2^/s) and BMN (5.5 × 10^–15^ m^2^/s) over ZIF-8. This corresponds to ∼100 and ∼200% enhancement in the diffusion of benzaldehyde and BMN respectively, in the mesoporous material. Additionally, the diffusivities obey the expected trend in molecular size (i.e., benzaldehyde diffuses faster than BMN) in both materials. However, the enhancement of diffusivity by M_ZIF-8 is greater for the bulkier molecule BMN (∼200% enhancement) than for benzaldehyde (∼100% enhancement). This difference in diffusivity increase can be attributed to the effect of molecular size/kinetic diameter on the diffusivity enhancement. This is consistent with the effect of the mesopores, which allow fast transport of both BMN and benzaldehyde. The above findings indicate that the faster catalytic kinetics in the hierarchical material M_ZIF-8 are largely due to enhanced intraparticle diffusion of reactants and products facilitated by the mesoporosity.

## Conclusions

Past demonstrations of hierarchical MOF synthesis were typically focused on mixed-linker, template-assisted, and non-uniform wet etching approaches. Here, we have explored an alternative strategy for the synthesis of hierarchical MOFs using the controlled generation of acid gas-induced defects. Our results suggest that SACRed, a versatile technique for introducing non-native linkers into MOFs, can be extended to the creation of hierarchical (mesoporous) MOFs by the introduction of non-native linkers amenable to a subsequent selective thermolysis. We demonstrated this concept by using SACRed to produce mesoporous ZIF-8 (M_ZIF-8). Solid-state double-quantum NMR spectroscopy provided considerable new insights into this process, e.g., revealing that the defects induced by acid gas exposure are preferentially clustered in ZIF-8. PXRD, N_2_ physisorption, water and 1-butanol adsorption measurements show that the microporous structure of ZIF-8 is retained while introducing large mesopores (∼50 nm) into the material, and that key chemical characteristics (such as hydrophobicity) associated with ZIF-8 are preserved in M_ZIF-8. Finally, we showed that M_ZIF-8 enhanced the rate of a catalytic reaction (condensation of malononitrile and benzaldehyde into benzylidenemalononitrile) over the conventional ZIF-8 material. We measured significant enhancements in reactant/product diffusivities in M_ZIF-8 owing to the mesoporosity, which is likely the main contributor to the observed enhancement in reaction rate. As mentioned earlier, the proven generalizability of SACRed to different MOFs creates potential for the synthesis of a variety of hierarchical MOFs. Similarly, this methodology is demonstrated for ∼10 μm crystals but could be tunable for a range of crystal sizes.

## Supporting Information Available

The Supporting Information document contains additional experimental data: SEM images of ZIF-8 and M_ZIF-8; fractional uptakes of benzaldehyde and benzylidenemalononitrile in ZIF-8 and M_ZIF-8; TGA curves of ZIF-8 and M_ZIF-8 and solution NMR measurements of M_ZIF-8 digested in CD_3_COOD (PDF)
