# Supplementary material for: Hierarchical ZIF-8 Materials via Acid Gas-Induced Defect Sites: Synthesis, Characterization, and Functional Properties
Source: ACS Appl Mater Interfaces. 2023 Aug 18;15(34):40623–32. doi: 10.1021/acsami.3c08344 (PMC10472435; doi:10.1021/acsami.3c08344)
Supplement: Supplementary file 1 — am3c08344_si_001.pdf [file am3c08344_si_001.pdf]

Supporting Information

for

Hierarchical ZIF-8 Materials via Acid Gas Induced Defect Sites: Synthesis, Characterization, and Functional Properties

Arvind Ganesan,[a] Johannes Leisen,[b] Raghuram Thyagarajan,[a] David S. Sholl,[a,c*] and Sankar Nair[a]*

[a]*School of Chemical & Biomolecular Engineering, Georgia Institute of Technology, Atlanta, GA 30332, USA*

[b]*School of Chemistry & Biochemistry, Georgia Institute of Technology, Atlanta, GA 30332, USA*

[c]*Oak Ridge National Laboratory, Oak Ridge, TN 37830, USA*

* Corresponding author: sankar.nair@chbe.gatech.edu; shollds@ornl.gov

Keywords: MOFs, acid gas, defects, hierarchical, Knoevenagel

Supporting Figures

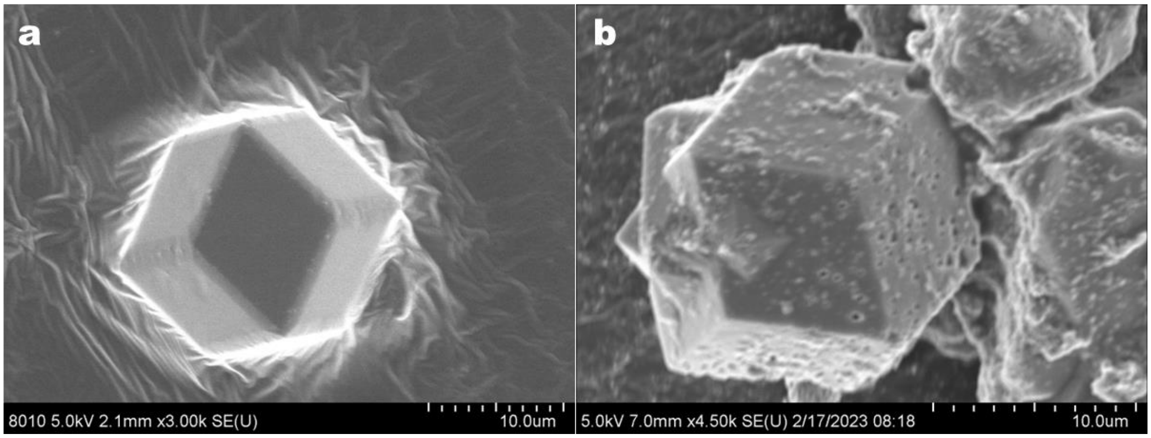

Figure S1. SEM images of **(a)** ZIF-8, and **(b)** M_ZIF-8.

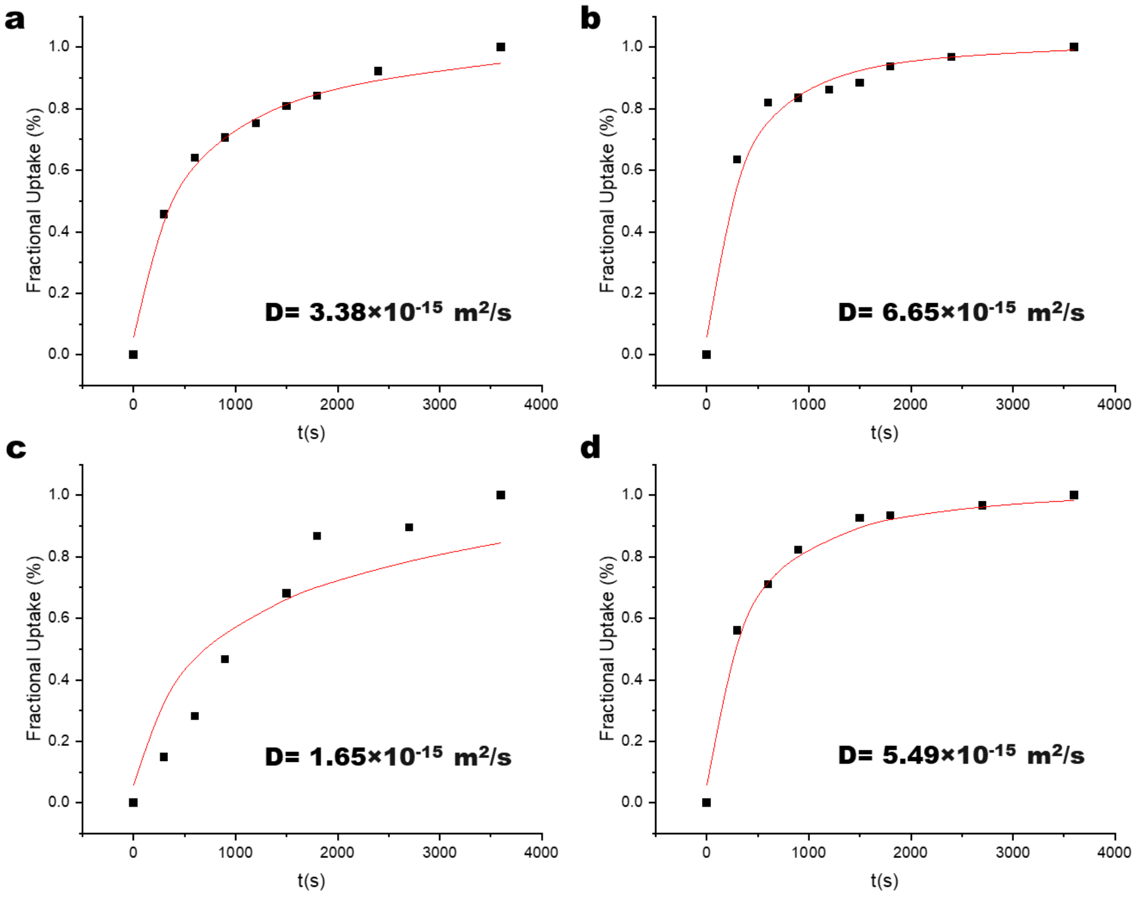

Figure S2. (symbols) fractional uptake of benzaldehyde in **(a)** ZIF-8 and **(b)** M_ZIF-8. and of benzylidenemalononitrile (BMN) in **(c)** ZIF-8 and **(d)** M_ZIF-8 respectively. The data are obtained by normalizing the absolute uptakes in **Figures 5b-5c** by the saturation values. The red curves show the fit of **Eq 1** with the Fickian diffusivity as the fitting parameter.

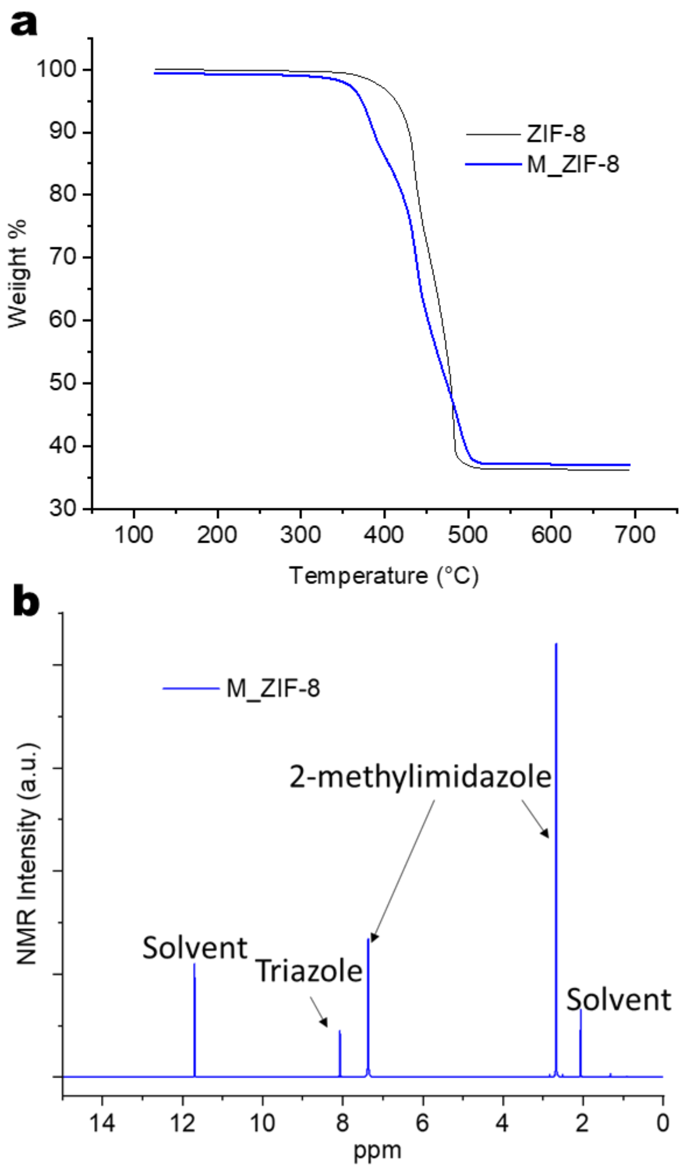

Figure S3. (a) TGA curves for ZIF-8, and M_ZIF-8 **(b)** solution NMR plot of M_ZIF-8 dissolved in CD_3COOD.
